# Invasive Streptococcus Pneumoniae Infection Causing Cardioembolic Stroke: A Clinical Proximity to Austrian Syndrome

**DOI:** 10.7759/cureus.1960

**Published:** 2017-12-18

**Authors:** Nilesh H Pawar, Oliver J Nickalls, Keng Leong Tan, Vui K Ho, Shin Yi Ng, Jiashen Loh

**Affiliations:** 1 Department of General Medicine, Sengkang General Hospital, Sengkang Health, Singhealth, Singapore; 2 Department of Radiology, Sengkang General Hospital, Sengkang Health, Singhealth, Singapore; 3 Department of Respiratory and Critical Care Medicine, Sengkang General Hospital, Sengkang Health, Singhealth, Singapore; 4 Department of Surgical Intensive Care, Sengkang General Hospital, Sengkang Health, Singhealth, Singapore; 5 Department of Surgical Intensive Care, Division of Anaesthesiology and Perioperative Medicine, Sengkang General Hospital, Sengkang Health, Singhealth, Singapore

**Keywords:** streptococcus pneumoniae, infective endocarditis, stroke, cardioembolic stroke, austrian syndrome, invasive pneumococcal disease

## Abstract

Streptococcus pneumoniae (S. pneumoniae) is a significant human bacterial pathogen and the major cause of pneumonia. In the post-antibiotic era, S. pneumoniae endocarditis is rare but carries a high risk of central nervous system (CNS) involvement. We present a case of invasive S. pneumoniae infection, which led to a cardioembolic stroke in a young man from septic emboli. Diagnosing a disseminated S. pneumoniae infection at an early stage is crucial and may improve patient outcomes.

## Introduction

Robert Austrian first described Austrian syndrome in 1957, which consists of the classic triad of pneumonia, endocarditis, and meningitis caused by Streptococcus pneumoniae (S. pneumoniae ), and commonly involves the aortic valve [1]. The affected patients are often debilitated or immunocompromised [1-2].

S. pneumoniae* *(the pneumococcus) is the most common cause of pneumonia in humans, but in the post-antibiotic era, cases of S. pneumoniae endocarditis are very rare [2-3]. We describe a case of cardioembolic stroke due to septic emboli from infective endocarditis caused by S. pneumoniae in an autistic adult.

## Case presentation

A 41-year-old Chinese man was admitted with a three-day history of fever associated with a nonproductive cough, lethargy, and poor oral intake. He was autistic and intellectually disabled with no medical history of note. He did not have any history to suggest asplenism or any infection with an encapsulated organism in the past. Premorbidly, he was wheelchair bound and required assistance in the activities of daily living. At presentation, he was uncommunicative and restless with a temperature of 38.5°C (101.3°F). His Glasgow Coma Scale (GCS) score was 11/15 (E4V2M5), blood pressure was 113/70 mm Hg, respiratory rate was 28 breaths/min, and oxygen saturation was 84% at room air. His condition deteriorated, and he subsequently required endotracheal intubation for airway protection and respiratory support. Cardiac auscultation revealed no murmur, whereas lung auscultation revealed bilateral crepitation. Abdominal examination was normal; he did not have any scars to suggest splenectomy. A complete neurologic assessment was not feasible, as the patient was uncommunicative and uncooperative. However, he was noted to be moving all four limbs equally.

The electrocardiogram was normal and did not show any heart block. The initial laboratory investigations showed a white blood cell count of 2.97 x 10^­­­9^/L (reference range, 4.0 to 9.0 x 10^9^/L) with 90.9% neutrophils, 5.4% lymphocytes, 2.7% monocytes, 0% eosinophils, and 1% basophils. His procalcitonin level was 3.4 µg/L (reference range, < 0.5 µg/L), and his C-reactive protein was 237 mg/L (reference range, 0.2 to 9.1 mg/L). Blood cultures were repeated five times since admission (on days 1, 2, 11, 12, and 17), and the result of each evaluation was negative. His urine was positive for the S. pneumoniae antigen (The test kit used to detect the urine S. pneumoniae antigen was Alere BinaxNOW (Alere, Waltham, Massachusetts, United States) Streptococcus pneumoniae Urinary Antigen Assay, which has a sensitivity and specificity of 86% and 94%, respectively). Oseltamivir 150 mg every 12 hourly was started empirically on admission. However, a few days later, his throat swab returned positive for Influenza A (method used: real-time multiplex polymerase chain reaction, Seegene Anyplex II RV16 Detection Kit - Version 1.1 (Seegene, South Korea)).

**Figure 1:**
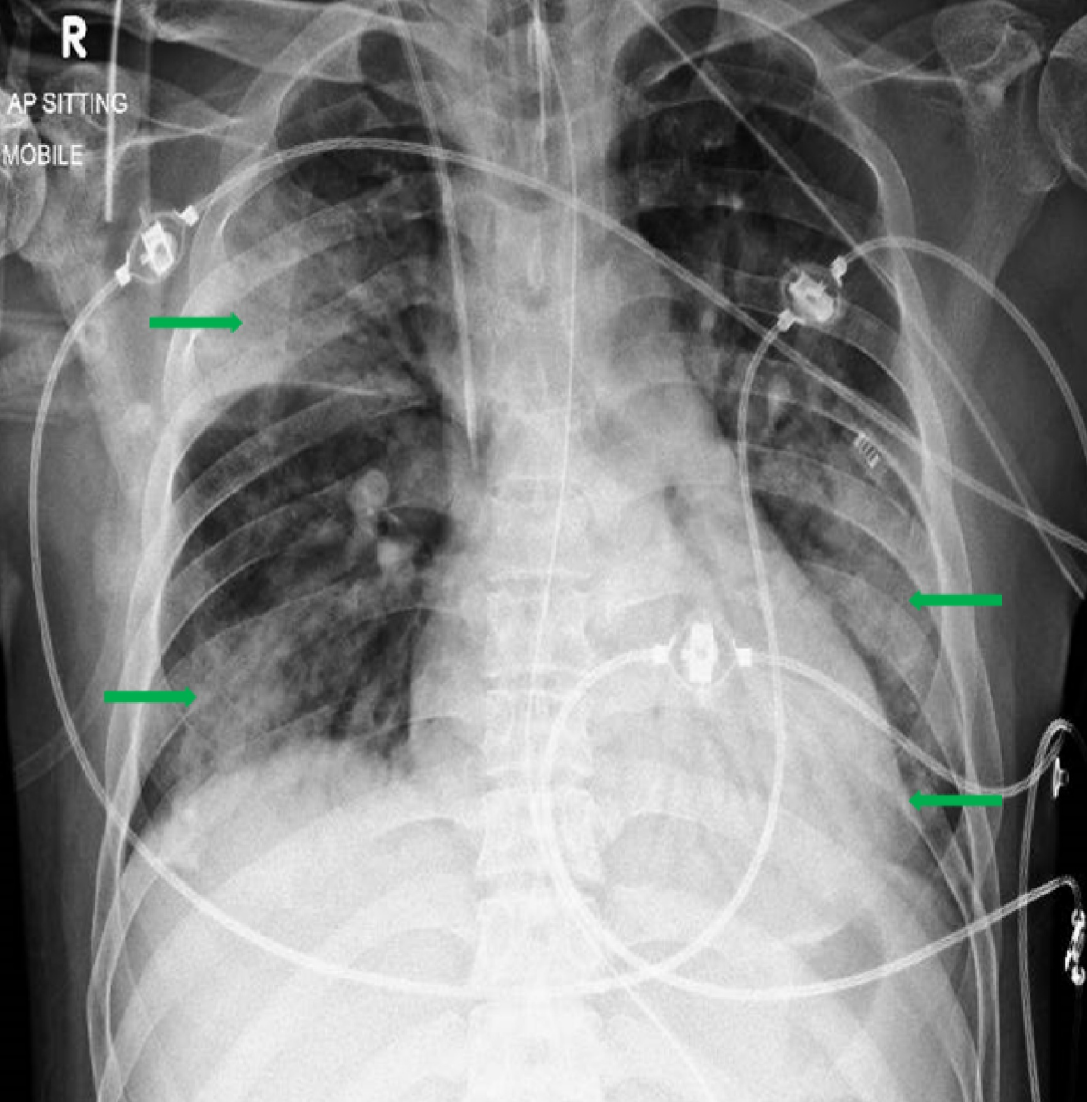
Chest radiograph on admission to the emergency department Extensive bilateral airspace changes (green arrows), involving the right upper and left middle zones and both lung bases

**Figure 2:**
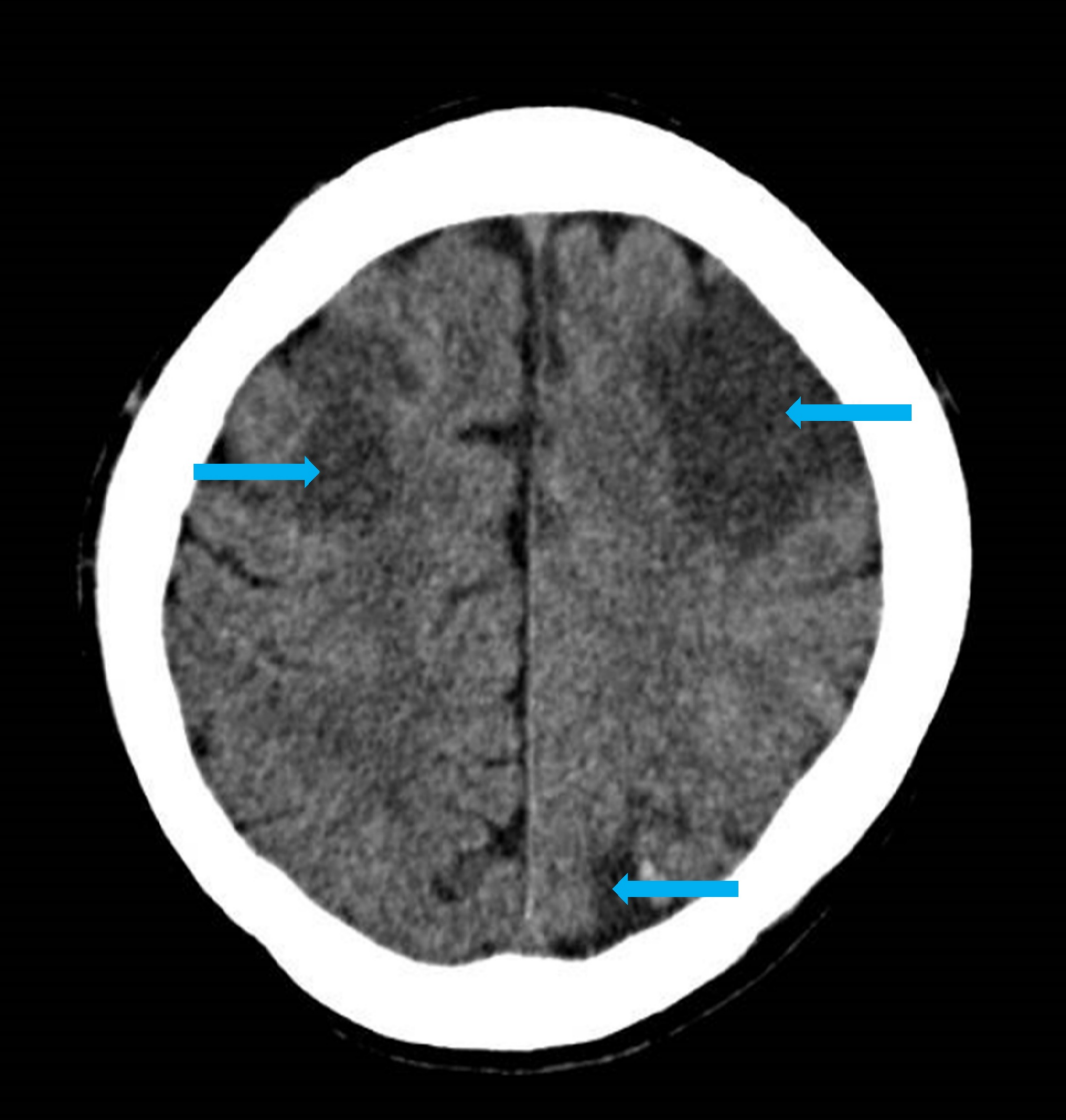
Computed tomography of the head done on day 15 after the admission Axial unenhanced computed tomography of the head shows a loss of grey-white matter differentiation and hypodensity (blue arrows) in the middle cerebral artery (MCA) territories bilaterally as well as in the left posterior parietal lobe compatible with acute infarcts

**Figure 3:**
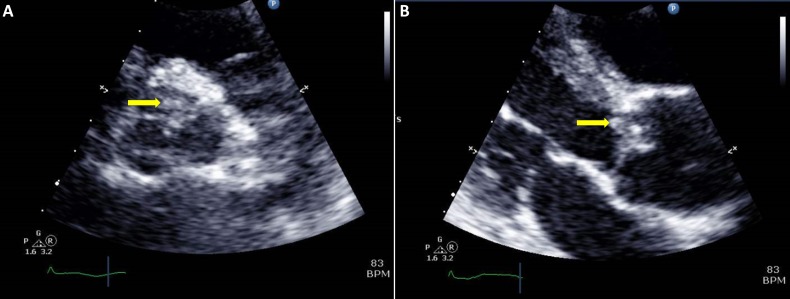
Transthoracic echocardiography Transthoracic echocardiography (A-B) showing tiny finger-like projections (yellow arrows) over the aortic valve, suggestive of vegetation over the aortic valve

**Figure 4:**
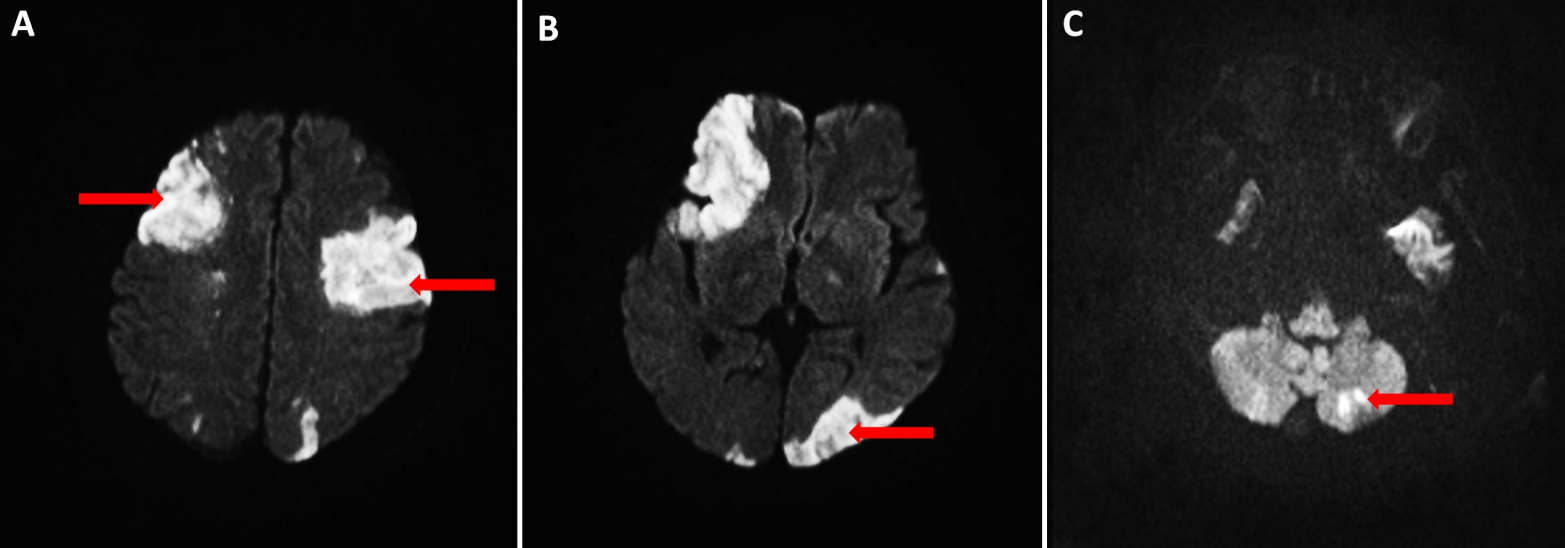
Magnetic resonance imaging of the brain (A-C) on day 18 after admission Axial diffusion-weighted images showing several areas of restricted diffusion (red arrows) within the middle cerebral artery territories bilaterally (A), in the left posterior cerebral artery territory (B), and in the left posterior inferior cerebellar artery territory (C), in keeping with acute infarct and suggestive of a cardioembolic etiology

A chest radiograph done on admission demonstrated bilateral areas of consolidation over his right upper and lower zones and the left midzone (Figure 1). Since admission to the intensive care unit, his Glasgow coma scale (GCS) was persistently low despite sedation breaks. Hence, on day 15, we obtained a computed tomography of the head (Figure 2), which demonstrated multiple hypodensities in both frontal lobes, the left parieto-occipital region, and, to a lesser extent, in the right parietal lobe and both cerebellar hemispheres. An urgent transthoracic echocardiogram (TTE) was obtained on day 16, which showed tiny finger-like projections on the right coronary cusps of the aortic valve, suggestive of aortic valve infective endocarditis (IE) and showed the thickening and prolapse of the aortic valve causing moderate aortic regurgitation (Figure 3). A magnetic resonance image (MRI) of his brain (Figure 4) was obtained on day 18, which showed multiple acute infarcts in the middle cerebral artery and cerebellar region.

He received intravenous (IV) tazocin (piperacillin/tazobactam) 4.5 g every eight hourly initially, which was changed to IV meropenem 2 g every eight hourly on the third day of admission and IV azithromycin 500 mg 24 hourly for three days. His condition worsened when he subsequently developed ventilator-associated pneumonia due to multidrug-resistant Acinetobacter baumannii and eventually died of respiratory failure 20 days after admission.

## Discussion

S. pneumoniae is a highly invasive, Gram-positive, extracellular bacterial pathogen that has the potential to cause an invasive infection resulting from the presence of bacteria in a normally sterile site [3]. The key to the diagnosis, in our case, was the presence of S. pneumoniae antigen in the patient’s urine, especially in the absence of positive blood cultures. Classically, an invasive S. pneumoniae infection is meningitis and bacteremia and is defined as an infection confirmed by the isolation of S. pneumoniae from a normally sterile site (e.g., blood or cerebrospinal fluid but not sputum). In our patient, we were not able to document bacteremia. This may be due to inadequate culture and sensitivity prior to the antibiotics exposure. The Streptococcus antigen in urine is 82% sensitive in detecting S pneumoniae bacteremia [4]. It also has a specificity of 97% [4]. We believe the presence of IE and central nervous system (CNS) septic emboli qualify this case as a case of invasive pneumococcal disease (IPD) [5]. His clinical course and poor response to treatment are consistent with IPD. Our patient had a possible concurrent Influenza infection, which is a known risk factor for IPD. It is important to raise awareness of the fact that failure to isolate S. pneumoniae on blood cultures should not give cause to exclude IPD unless the patient’s course is clearly improving or an extensive evaluation is completed. 

An MRI brain performed on day 18 showed restricted diffusion in multiple vascular territories, suggesting a cardioembolic etiology, a consequence of septic embolism from IE. The diagnosis of IE is usually delayed due to the late occurrence of cardiac murmur and other classical signs of endocarditis [1-2]. It is interesting to note that although mitral valve lesions are more commonly known to embolize [6], our patient had an aortic valve vegetation. There is a possibility that our patient had mitral valve as well as aortic valve vegetation and mitral valve vegetation had embolized. We postulate that the emboli happened after admission, as the GCS was stable on admission and dropped only later.

The MRI brain dated the infarct to have occurred approximately 10 to 13 days after his admission [7]. Most embolizations usually occur within the first two to four weeks of initiation of antibiotics, though it can also occur before the diagnosis has been made [8]. Transesophageal echocardiography (TEE) is more sensitive than TTE in the detection of vegetations [9], but our patient was very sick and the cardiologist opined that the added value of TEE was minimal. In cases of negative TTE and a high suspicion for IE, TEE should be performed [9].

The diagnosis was delayed in this case because the patient was autistic, uncommunicative, and almost immediately intubated upon admission because of his respiratory failure. In the absence of a positive blood culture, his minimum inhibitory concentration of penicillin against S. pneumoniae could not be determined. This would have an important implication in the management of IE and CNS septic emboli. Although steroid use can offer a mortality benefit in a subset of patients with S. pneumoniae meningitis [10], the need for early administration means the window of steroid use is usually missed in patients with Austrian syndrome, in which the diagnosis is often delayed. Also, our patient did not present in the appropriate GCS group (8-11), which has been found to benefit most from steroids [10]. The overall incidence of the invasive pneumococcal disease has declined following the introduction and routine childhood immunization with the 7-valent (PCV-7) and, later, the 13-valent pneumococcal conjugate vaccines (PCV-13). Our patient did not benefit from the childhood pneumococcal conjugate vaccination, as this was introduced later [3].

Even in the post-antibiotic era, a subset of patients with poor access to or a poor ability to seek medical care promptly may be in a setting not too different from the pre-antibiotic era. Although our case does not fulfill the classical triad of Austrian syndrome, we wish to highlight the possibility of IE and its attendant implications in patients with S pneumoniae pneumonia. A convenient differential diagnosis in this cohort presenting with seizures would be penicillin-related toxicity, which should be only a diagnosis of exclusion.

## Conclusions

S. pneumoniae is a common human bacterial pathogen. IE secondary to S. pneumoniae is rare but may occur in debilitated patients. With this case, we would like to raise awareness that even in the post-antibiotic era, patients who have poor access or a poor ability to seek medical care promptly are not too different from patients in the pre-antibiotic era. Thus, a high index of suspicion and early diagnosis is crucial to achieve better outcomes for such patients.
